# Direct and indirect costs of diabetes mellitus in Mali: A case-control study

**DOI:** 10.1371/journal.pone.0176128

**Published:** 2017-05-18

**Authors:** Clara Bermudez-Tamayo, Stéphane Besançon, Mira Johri, Sidibe Assa, Jonathan Betz Brown, Kaushik Ramaiya

**Affiliations:** 1 Andalusian School of Public Health, Granada, Spain; 2 CIBER Epidemiologia y Salud Publica (CIBERESP), Instituto de Salud Carlos III, Madrid, Spain; 3 NGO Santé Diabète, Bamako, Mali; 4 Centre de Recherche du Centre Hospitalier de l'Université de Montréal (CRCHUM), Montréal, Canada; 5 Département d'administration de la santé, École de santé publique, Université de Montréal, Montréal, Canada; 6 Endocrinology Department, Mali National Hospital, Bamako, Mali; 7 Kaiser Permanente Center for Health Research, Portland, Oregon, United States of America; 8 Shree Hindu Mandal Hospital, Dar es Salaam, Tanzania; King Abdullah International Medical Research Center, SAUDI ARABIA

## Abstract

**Background:**

Diabetes mellitus (DM) is one of the most burdensome chronic diseases and is associated with shorter lifetime, diminished quality of life and economic burdens on the patient and society as a result of healthcare, medication, and reduced labor market participation. We aimed to estimate the direct (medical and non-medical) and indirect costs of DM and compare them with those of people without DM (ND), as well as the cost predictors.

**Methods and findings:**

Observational retrospective case–control study performed in Mali.

Participants were identified and randomly selected from diabetes registries. We recruited 500 subjects with DM and 500 subjects without DM, matched by sex and age. We conducted structured, personal interviews. Costs were expressed for a 90-day period. Direct medical costs comprised: inpatient stays, ICU, laboratory tests and other hospital visits, specialist and primary care doctor visits, others, traditional practitioners, and medication. Direct non-medical costs comprised travel for treatment and paid caregivers. The indirect costs include the productivity losses by patients and caregivers, and absenteeism. We estimate a two-part model by type of service and a linear multiple regression model for the total cost. We found that total costs of persons with DM were almost 4 times higher than total cost of people without DM. Total costs were $77.08 and $281.92 for ND and DM, respectively, with a difference of $204.84.

**Conclusions:**

Healthcare use and costs were dramatically higher for people with DM than for people with normal glucose tolerance and, in relative terms, much higher than in developed countries.

## Introduction

Over the last two decades the global health landscape has undergone rapid transformation with substantial variation across regions and countries. Nowhere is this contrast more striking than in Sub-Saharan Africa. Progress has been made in reducing communicable diseases and early childhood conditions; these diseases still account for the highest health loss in the region but their relative burdens are lower. However, disease burden from non-communicable causes has increased and currently accounts for 20% of deaths, particularly diabetes (DM), stroke, depression, and ischemic heart disease [1].

According to estimates the prevalence of DM will continue rising by 98% during the next 20 years in Africa, with dramatic implications for public health and national budgets of the poorest countries [1, 2]. More than 21 million people have DM and this figure will almost double by 2035 if conditions and trends do not change. On top of this, Africa has the highest percentage (62%) of undiagnosed people, who are at higher risk of developing harmful and costly complications [3].

DM imposes a considerable burden on health systems and societies, leading to a variety of disabling, life-threatening and expensive complications such as cardiovascular disease, retinopathy, neuropathy, and nephropathy. The cost implications of DM for society are multi-level: direct costs for people with DM, their families and the healthcare sectors, and indirect costs to society and government [4]. Costs relating to loss of productivity are exacerbated in Sub-Saharan Africa because working-age adults account for a high proportion of the DM burden.

This scenario becomes more complex because of weak health systems, limited access to services or use of substandard services [3] and the high risk of catastrophic expenditure, which means very high healthcare spending in relation to income beyond which an individual begins to sacrifice items of basic consumption. The financial catastrophe is predicted by the presence of three conditions [5]: healthcare cost paid out of pocket, individuals’ inability to pay and absence of prepayment mechanisms to pool financial risks.

Little is known about the economic impact of diabetes in low and middle-income countries (LMIC). A recent review by Seuring et al [6] found evidence of a particularly strong and direct economic impact of DM, but available studies have been confined almost exclusively to high-income countries (HIC) [7]. Estimates of the economic burden can assist decision-makers in understanding the magnitude of the problem, prioritizing research efforts, planning resource allocation properly to manage the condition and enabling health systems better to prepare to meet population health needs. Disease cost estimates also help prioritize interventions, which must be done in the context of limited healthcare resources [7].

The purposes of this study were to elucidate the direct and indirect costs of DM and their predictors, based on a comprehensive case–control study in Mali. Mali was chosen for this study as, like many other countries in Sub-Saharan Africa, it faces a growing burden of diabetes due to increases in urbanization, the challenges of nutritional transition and increasingly sedentary behavior [8].

## Materials and methods

### Study design and sample

Observational retrospective research carried out as a case–control study, comparing the direct and indirect costs of people with DM and those of people without the disease (non-diabetic group, ND). The study was completed in Mali and was part of a larger study performed in four Sub-Saharan African countries–Cameroon, Mali, Tanzania, and South Africa–and described in detail elsewhere [9].

Mali has a population of 14.8 million people and a life expectancy at birth of 57 years for men and women [1]. Total expenditure on health per capita per year is Int. $ 74, representing 5.8% of total Gross Domestic Product. The prevalence of diabetes in Mali is estimated at 1.28% of the adult population, although local experts would state that this seriously underestimates the true burden [8].

**Fig 1:**
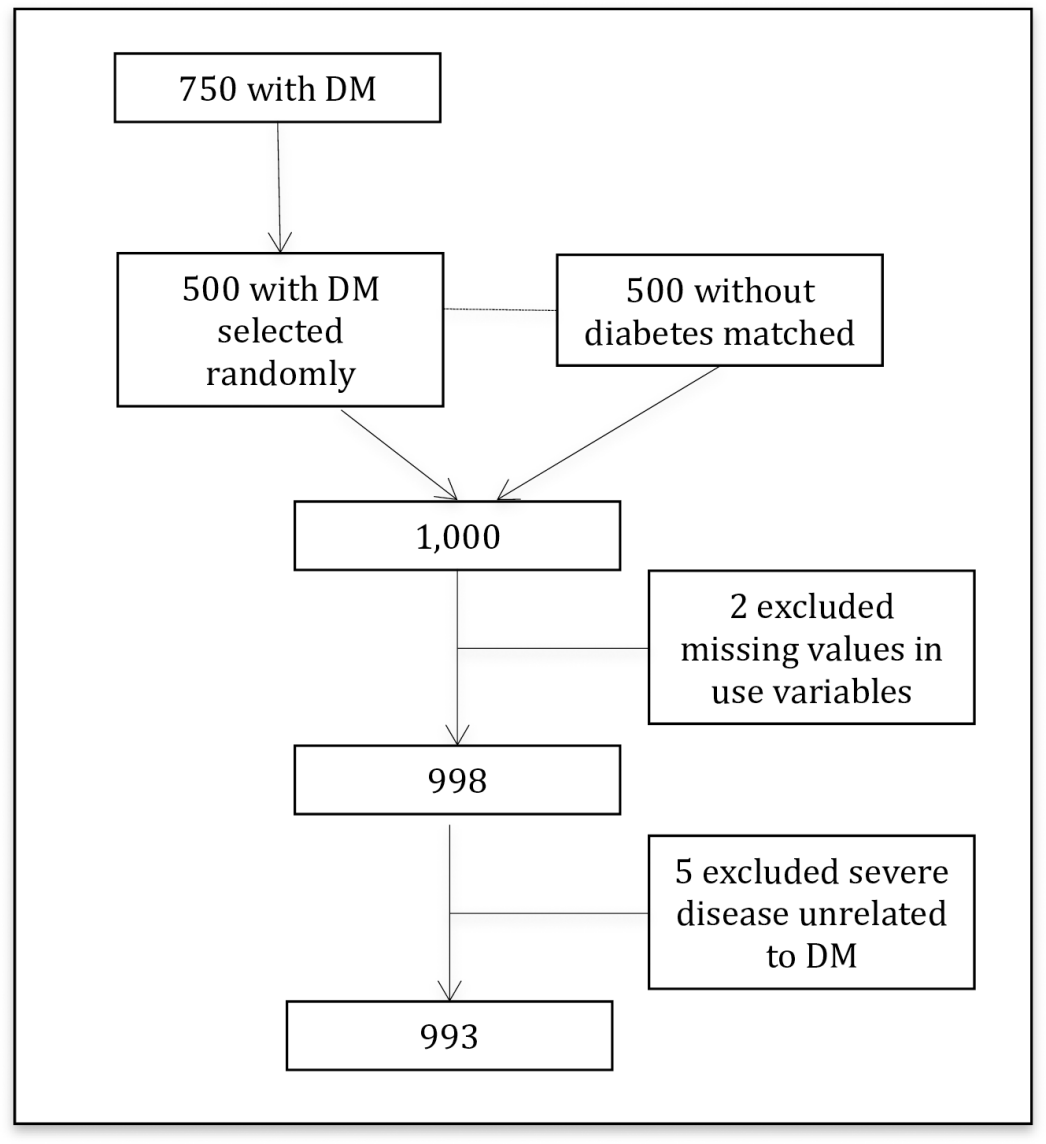
Flowchart of sample selection process.

The sample included 500 cases and 500 controls to provide 90% power to detect a 5 percentage point difference in rates and proportions between cases and controls in each country, based on the differences in expenditure values in the worst case (50%). Thanks to the previous creation of diabetes registers drawn from diabetes clinic records, the recruitment pool included all persons with diagnosed diabetes living in or near the country’s three largest cities: Bamako, Sikasso and Timbuktu. We drew up a random list of 750 people from these registers. 500 people were randomly selected from the list and when a person refused to participate, another one on the list was selected at random. In total, 65 people refused to participate. Once cases were identified and agreed to participate, each case was asked to identify five persons of the same sex and approximate age living closest to them. We then contacted and recruited one control subject for each case using this information. Potential controls who said they had been diagnosed with diabetes were excluded. From this population, 5 cases were excluded because of their expenditure on severe diseases unrelated to diabetes and 2 for whom values of resources use were missing. (Fig 1)

### Data collection and resource identification

To collect data we used a questionnaire conducted by an interviewer. To improve accuracy of recall during the interview, we asked about events occurring only during the previous 90 days. The questionnaire included questions about demographics and socioeconomic characteristics, the use of allopathic and traditional medical services and payments made at time of service for medical care services. The questionnaire was published elsewhere [9]. Interviewers were medical professionals or field workers who were trained and had performed practice interviews prior to entering the field. Before starting the interview, interviewers confirmed and recorded each subject’s informed voluntary consent to participate. The interviews were completed in 2008–2009 and took place in subjects’ homes.

### Estimated costs and resource valuation

Direct (medical and non-medical) and indirect costs were estimated for each group and are expressed for a 90-day period.

Direct medical costs comprised the costs relating to: 1) services provided by or at an hospital, including inpatient stays, ICU stays, laboratory tests and other hospital visits; 2) services provided outside hospitals: specialist and primary care doctor visits, community health worker, nurse, health educator, pharmacist; 3) traditional practitioners and 4) medication.

Direct non-medical costs comprised: 1) cost of travel for treatment -to and from hospitals, waiting for admission, and during institutionalized treatment- and 2) paid caregivers. Traditional practitioners' visits, medication and direct non-medical costs were assessed according to self-reported data by subjects. Other cost components were assessed as described in Table 1.

**Table 1:** Data used for estimating indirect and direct costs of diabetes in Mali.

Item	Int. $	Source of data, year
Cost per bed day by hospital level		WHO-CHOICE unit cost estimates for service delivery, 2009
Primary	7.49	
Secondary	7.81	
Tertiary	10.10	
Cost per bed ICU	33.04	
Cost per outpatient visit by hospital level		
Health Centre (no beds)	2.14	
Health Centre (with beds)	2.64	
Primary-level hospital	3.01	
Secondary-level hospital	3.14	
Daily GNI per capita (PPP)	= 570 Int. $/365 days = 1.56	World Bank, 2009

The indirect costs comprised opportunity cost of time lost due to morbidity and include productivity losses by patients and by relatives or caregivers accompanying patients, as well as absenteeism. The human capital approach was adopted to estimate indirect costs. This method assumes that the value of lost work is equal to the amount of money which the individual would have been paid to do the work in question.

ICn (Indirect costs) = (CTDn + CPVn), where CTDn is the total cost of productive time lost due to diabetes-related temporary disability and CPVn is the productivity loss due to work time lost by relatives accompanying and visiting patients. The cost of disability in those retired (over 60 years old) was ignored, given that the human capital approach values costs using market earnings (10). Unit cost for valuation is described in Table 1.

### Data analysis

Descriptive statistics are presented as percentages and mean with standard deviation. Group comparisons for continuous variables were performed by the Student t-test. The Chi- squared statistic was used to test differences between proportions, and the Fisher test when one or more cells had an expected frequency of five or less.

We report the mean (SD) of resources and standard deviations for direct costs per case in each group. Since variables were highly skewed, we used bootstrap estimation with 1,000 re-samples to obtain an adjusted standard deviation for average cost [11] and clustered by site. We also used estimation in addition to a standard t-test on the mean difference in cost.

#### Direct and indirect costs

We estimate separately models of services provided by or at hospital, services provided outside hospitals, non-medical costs, medication and indirect costs to measure differences in the relationship between DM and ND. As independent variables, we also considered 1) socioeconomic characteristics: age (< = 50, > 50), sex, level of education (primary or lower, secondary or higher), income, and 2) health-related characteristics: time suffering from DM and communicable diseases. Lastly, we considered the interactions of income, time suffering from DM and DM.

Given the nontrivial proportion of subjects without cost (zero mass and skewed outcomes), the results were estimated using a two-part model [for details on this approach, see Madden [12] and Belloti [13]]. The two-part model estimates the probability of any cost and the level of non-zero costs among subjects separately. The probability model is estimated using logistic regression and the conditional logged costs model is estimated using ordinary least squares. We use group-specific smearing factors based on individual residuals to retransform the results to the original scale and employ bootstrapping methods to estimate the standard errors from the two-part model. We use Duan’s [14] smearing estimator.

#### Total costs

The model for total cost was estimated by a multiple linear regression and its estimation using ordinary least squares. The goodness of fit was determined through the corrected R2 value and the F Snedecor, mean squared error, and for each of β coefficients. The assumptions of normality, homoscedasticity and linearity were considered. Split analysis validation was carried out to avoid overfitting. As dependent variable we studied total cost and as independent variables we considered DM and socioeconomic and health-related characteristics.

Because there is no suspected seasonality in expenditure, the annual cost arising was calculated by multiplying the cost of the previous 90 days by 4. We performed analyses using a definition consistent with previous studies: household out-of-pocket medical spending $ 40% of capacity to pay [15].

Statistical analyses were done using STATA SE 14.1.

#### Ethics statement

Approval was given by the Research Ethics Committee of the Faculty of Medicine, Pharmacy and Dentistry (University of Bamako). Patients gave written informed consent and the research was conducted in accordance with the declaration of Helsinki.

## Results

Seventy-five percent of subjects were women with an average age of 52.3 ± 0.5 years. Subjects with DM were similar to ND group subjects in most characteristics (Table 2); sex, age, site, level of education completed and monthly income.

**Table 2:** Characteristics of the study population.

Variable	ND n = 496	DM n = 497	P value
**Demographic and socioeconomic**			
Female, n (%)	374 (75.4)	373 (75.1)	0.898*
Age, Mean (SE)	51.3 (0.5)	52.6 (0.5)	0.052**
Family size, Mean (SE)	13.8 (0.4)	15.7(0.5)	0.003**
Site, n (%)			
Bamako	298 (60.1)	298 (60.0)	0.999*
Sikasso	148 (29.8)	149 (29.9)	
Timbuktu	50 (10.1)	50 (10.1)	
Level of education completed, n (%)			
Secondary or lower	406 (81.9)	424 (85.3)	0.141*
Secondary or higher	90 (18.1)	73 (14.7)	
Current work situation, n (%)			
Employed	295 (61.2)	201 (43.2)	<0.001*
Not working due to ill health	3 (0.6)	35 (7.5)	
Not working due to other reasons, or retired	33 (6.9)	68 (14.6)	
Homemaker	151 (31.3)	161 (34.6)	
Monthly family income (US$)	76.6 (5.6)	72.8 (6.3)	0.648**
Sources of payment for medical care, n (%)			
Paid from own resources (self-income, savings, money borrowed, sale of possessions)	397 (81.0)	275 (55.4)	<0.001***
Paid from social support funds or donations	5 (1.0)	3 (0.6)	
Paid with money from family or friends	88 (18.0)	218 (44.0)	
**Disease history**			
Cardiovascular events, n (%)	12 (2.4)	54 (10.8)	<0.001***
Pulmonary diseases, n (%)	43 (8.7)	57 (11.5)	0.143
Lower extremity problems (amputation of a toe, foot, or leg; peripheral neuropathy; and/or history of foot or leg ulcer), n (%)	68 (13.7)	282 (56.7)	<0.001**
Eye disease (including laser treatment or other eye surgery), n (%)	31 (6.3)	84 (16.9)	<0.001**
Communicable diseases, n (%)	305 (61.6)	350 (70.4)	0.003
Years since DM diagnosis, n (%)	-	5.6 (0.2)	-
**Health status**			
Bad, passable	131 (26.5)	328 (66.3)	<0.001*
Excellent, very good, good	364 (73.5)	167 (33.7)	

*Chi^2^

**Student t

***Fisher

Subjects in the two groups were in different current work situations (employed 61.2% and 43.2% for ND and DM, respectively) and they reported different sources of payment for medical care (self-paid 81.0% and 55.4% for ND and DM, respectively). Family size, defined as ‘‘the number of people who normally eat with you where you live,” was also different between groups (larger among DM than among ND).

Persons living with diabetes had more health problems than neighbors of the same age and sex in terms of cardiovascular events (2.4% and 10.8% for ND and DM, respectively), lower extremity problems (13.7% and 50.7% for ND and DM, respectively), eye disease (6.3% and 16.9% for ND and DM, respectively) and communicable diseases (61.6% and 70.4% for ND and DM, respectively). Self-reported health was also different between groups (excellent or good 33.7% and 73.5% for DM and ND, respectively). Table 2 summarizes the demographic, socioeconomic and health features of the sample.

### Direct and indirect costs

The main areas of direct costs (inpatient stays, ICU stays, other hospital visits, laboratory tests, specialist and primary care doctor visits, others-community health worker, nurse, health educator, pharmacist-, traditional practitioners, medication, travel costs, paid caregiver), as well as indirect costs (productivity loss, absenteeism, productivity loss caregiver) are summarized in Table 3. There is a description of the average cost for DM and ND groups and of the differences between the groups.

**Table 3:** Total resource use and direct costs per person (last 90 days) for the entire population. (int $) (unadjusted).

Type	Cost ND		Cost DM		Cost Diff	P value
	Mean (SE)	n	Mean (SE)	n	(ND-DM)	
**Services provided by or at Hospital**						
Inpatient stays	0.6 (0.5)	6	4.52 (1.4)	26	+3.9 (1.4)	0.008
ICU stays	3.0 (0.9)	3	9.8 (4.6)	15	+6.8 (4.3)	0.109
Other hospital visits	1.8 (0.4)	149	14.8 (3.9)	448	+13.0 (0.9)	<0.001
Laboratory tests	7.8 (2.5)	113	42.1 (8.3)	447	+34.3 (1.7)	<0.001
*Total*	11.5 (2.0)	149	69.3 (15.9)	448	+57.8 (5.6)	<0.001
**Services provided outside hospitals**							
Specialist and primary care doctor visits	0.2 (0.1)	13	0.2 (0.1)	22	+0.002 (0.1)	0.977
Others (community health worker, nurse, health educator, pharmacist)	0.3 (0.1)	14	0.4 (0.2)	40	+0.3 (0.1)	0.004
Traditional practitioners	1.39 (0.4)	114	0.80 (0.6)	60	-0.60 (0.3)	0.045
*Total (excluding* traditional practitioners)	0.3 (0.1)	26	0.6 (0.3)	53	+0.3 (1.1)	0.020
**Direct non-medical costs**							
Travel costs	0.2 (0.1)	73	1.3 (2.1)	74	+1.2 (0.5)	0.038
Paid caregiver	0.2 (0.1)	3	0.5 (0.2)	7	+0.3 (0.2)	0.129
*Total*	0.3 (0.2)	75	1.8 (2.1)	78	+1.47 (0.6)	0.013
**Medication**	3.6 (0.5)	183	13.8 (2.3)	441	+10.2 (0.9)	<0.001
***Total direct costs***	23.4 (2.9)	318	127.2 (6.4)	488	+75.3 (3.9)	<0.001
**Indirect costs**						
Productivity loss	39.8 (9.2)	90	101.5 (17.8)	238	+61.7 (7.3)	<0.001
Absenteeism	18.7 (3.6)	128	48.5 (7.6)	219	+29.8 (4.8)	<0.001
Productivity loss caregiver	7.1 (3.4)	32	36.7 (14.8)	158	+29.6 (3.1)	<0.001
*Total*	65.65 (6.2)	158	186.8 (9.5)	316	+121.2 (11.4)	<0.001
***Total costs***	88.7 (12.1)	368	314.8 (45.9)	491	+226.1 (13.8)	<0.001

People with DM reported more use, direct and indirect costs than people without DM for all the areas studied (statistically significant), except for traditional practitioners.

Table 4 presents the estimated coefficients and standard errors from the logistic and conditional cost models. The results reveal that the odds of using inpatient services correlate positively with DM, age, communicable diseases, income (for subjects with DM), and time suffering from DM. The model explains the 36% variability. A similar pattern was found for the level of cost; positively correlated with DM, age, income, and time suffering from DM, communicable diseases. (The model explains 36% and 31% variability for the first and the second part, respectively).

**Table 4:** Impact of DM on direct and indirect costs (previous 90 days). Results From Two-Part Model.

Characteristics	Services provided by or at Hospital		Services provided outside hospitals		Medication		Indirect costs	
	Prob. of any Cost	Level of Cost*	Prob. of any Cost	Level of Cost	Prob. of any Cost	Level of Cost	Prob. of any Cost	Level of Cost
Intercept	-1.589***	2.342***	-1.230***	1.448***	-0.728***	1.602***	-0.932***	4.893***
DM								
No	Reference							
Yes	2.800***	1.316***	1.137***	1.708***	2.686***	0.550***	1.278***	0.434***
Age								
< = 50	Reference							
> 50	0.596**	0.112**	0.598**	0.009**	0.126**	0.138**	0.096*	-0.139*
Sex								
Female	Reference							
Male	0.180	0.037	0.233	0.265	0.312*	0.048	0.085*	0.236*
Level of education								
Primary or lower	Reference							
Secondary or higher	-0.034	0.153	-0.032	0.314	-0.385*	0.035	-0.031**	-0.196
Income	0.001	0.002**	0.001	-0.002**	0.001	0.001	-0.001**	0.001
Time with DM	3.048	0.225**	3.583	0.261**	0.191	0.090	-3.090	0.019
Communicable diseases								
No	Reference							
Yes	1.057***	0.285**	0. 578	0.474**	0.319*	0.016	0.369	-0.081
**Interactions**								
Income X DM	0.001*	0.002	0.002***	0.002**	0.001	0.001	0.001***	0.001
Time with DM XDM	3.363*	0.220**	3.594	0.251**	0.169	0.081	3.082	0.001**
Adj R-squared/ Pseudo R2	0.360	0.313	0.326	0.289	0.330	0.168	0.083	0.067

*** Statistical significance at the 1-percent level.

** Statistical significance at the 5-percent level.

* Statistical significance at the 10-percent level.

The odds of using outpatient services are also significant and correlate positively with DM, age, and income (for subjects with DM). Cost of using these services was significant and correlates positively with DM, age, income, time suffering from DM and communicable diseases. (The model explains 33% and 29% variability for the first and the second part, respectively.)

The odds of using medication are significant and correlate positively with DM, age, being male, level of education (secondary or higher) and communicable diseases. Cost of using medication was significant and correlates positively with DM and age. (The model explains 33% and 17% variability for the first and the second part, respectively.)

The findings also indicate that the odds of indirect costs correlate positively with DM, age, being male, level of education, and income. Indirect costs was significant and correlates positively with DM, age, being male and time suffering from DM. (The model explains 8% and 7% variability for the first and the second part, respectively.)

Table 5 lists the results of the multivariable regression model for the total costs. Total cost correlates positively with age, communicable diseases, and time suffering from DM.

**Table 5:** Impact of DM on total costs.

Variable		Coefficient	P Value
Intercept		5.180	0.001
DM	No	1	0.001
	Yes	4.150	
Age	< = 50	1	0.031
	> 50	0.098	
Sex	Female	1	0.379
	Male	0.474	
Level of education	Primary or lower	1	0.748
	Secondary or higher	-0.202	
Income		0.003	0.234
Time with DM		0.578	0.296
Communicable diseases	No	1	0.001
	Yes	1.631	
Income X DM		0.001	0.788
Time with DM XDM		-0.480	0.048
Adj R-squared		0.2810	

The marginal effect of DM is described in Table 6. Costs of services provided by or at Hospital were $9.39 and $74.95 for ND and DM, respectively, with a difference of $65.56 (8 times higher). Costs of services provided outside hospitals were $0.94 and $2.05 for ND and DM, respectively, with a difference of $1.11 (2.2 times higher). Costs of medication were $3.43 and $14.37 for ND and DM, respectively, with a difference of $10.94 (4.2 times higher among persons with DM than among persons without DM). Regarding indirect costs, DM had a mean cost of $190.5 and ND had a mean cost of $63.3 (a difference of $127.2), equivalent to 3 times higher. Total costs were $77.08 and $281.92 for ND and DM, respectively, with a difference of $204.84, equivalent to 3.7 times higher.

**Table 6:** Incremental costs for the combined version of the two-part model for DM (previous 90 days).

	ND* n = 496 Mean, CI 95%			DM* n = 497			Diff.*	% Diff.	P value
Services provided by or at Hospital	9.39	7.16	11.62	74.95	66.47	83.44	65.56	7.98	0.001
Services provided outside hospital	0.94	0.32	1.56	2.05	0.70	3.39	1.11	2.18	0.021
Medication	3.43	2.65	4.21	14.37	12.45	16.29	10.94	4.19	0.001
Indirect costs	63.32	47.50	79.13	190.55	158.95	222.14	127.23	3.01	0.001
Total costs	77.08	57.63	96.52	281.92	238.57	325.26	204.84	3.86	0.001

*All costs given in 2013 $int = (Malian franc = 0.0021 $int) (World Bank, 2015)

The average yearly direct cost was $55.04 ($40.52-$69.56) for ND patients and $365.48 ($318.48-$412.48) for DM patients. The yearly average for indirect cost was $253.28 ($190.00-$316.52) and $762.20 ($635.80-$888.56) for DM patients. Lastly, the total annual cost was $308.3 ($230.5-$386.1) for ND patients and $1,127.7 ($954.3-$1301.0) for DM patients. Diabetic individuals have a higher likelihood of incurring catastrophic medical spending (10.48 vs. 56.34%; difference 45.8% [95% CI 32–57.7]) compared with otherwise similar individuals without diabetes.

**Fig 2:**
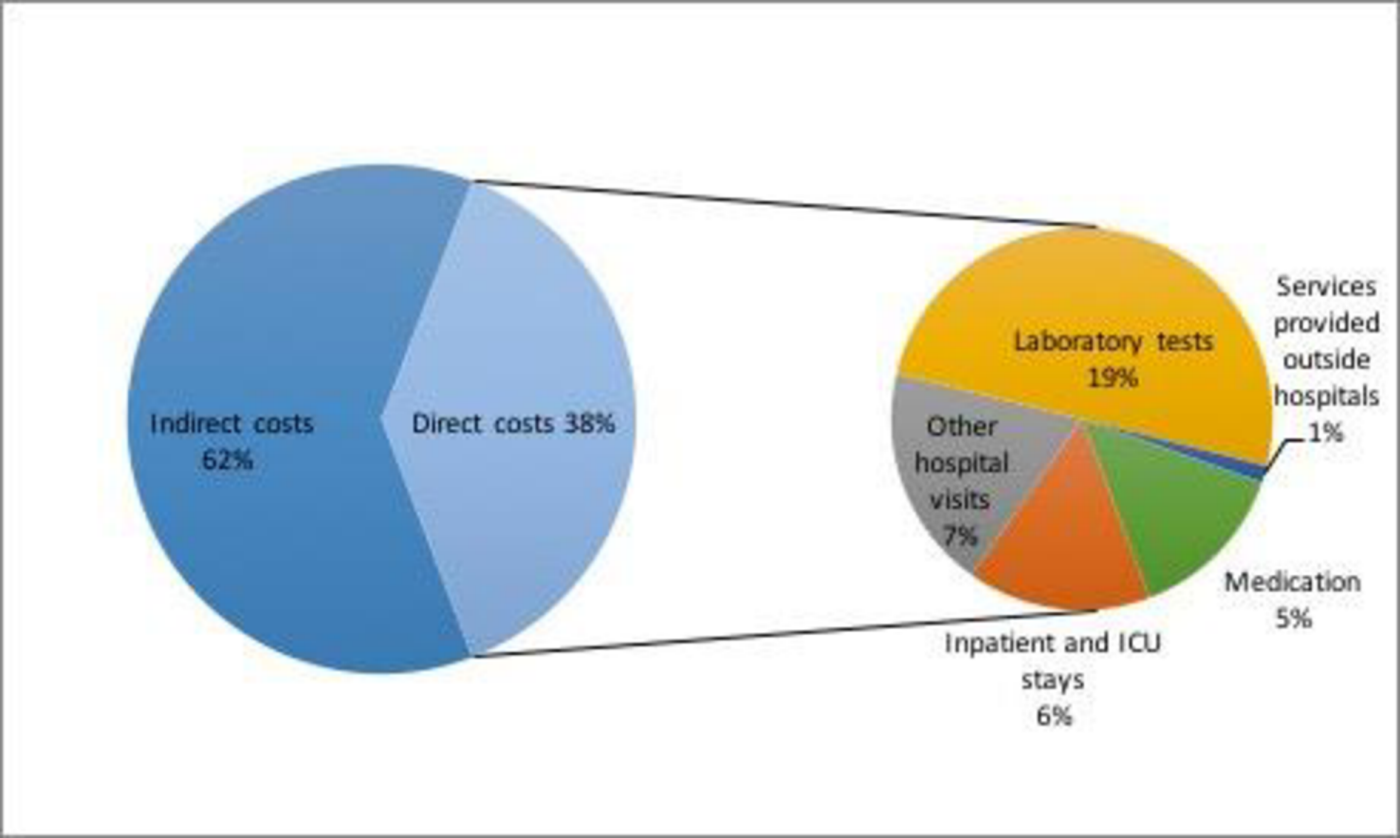
Components of cost attributable to DM.

The relative distribution of total attributable costs by components is described in Fig 2. 39% of costs of DM were direct costs and 61% were indirect costs. The most significant components of direct cost were laboratory tests (19%) and other hospital visits (7%).

## Discussion

The study provides relevant information in an area where studies are scarce. There are few studies on cost of diabetes care that have been carried out in the region [16–21] and moreover, this work is the first of its kind performed in Sub-Saharan Africa that considers a control group for estimating costs attributable to diabetes, as recommended in guidelines [22].

We found that the total costs of persons with DM were almost 4 times higher than the total cost of people without diabetes. This is higher than the figure published in a study performed in China using a similar methodology, where the estimate of cost among persons with DM was 3.4 times higher than among persons without DM [23]. This is also higher than the results of a study in Argentina, where total medical costs were 3.6 times higher in persons with DM compared with persons without DM [24]. The ratio obtained in this study is higher than when performed in developed countries, where the ratio is between 2.0 and 2.4 [25–27]. Its size suggests that the human and economic impact of DM might be much greater in LMIC than in developed countries. In Africa, testing for DM usually occurs after patients present with complications known to arise from DM. This means that persons recognized as having DM in Africa are much sicker and need more services and medication than their counterparts in other places. In addition, Africans with DM are not given medicines to control hypertension and dyslipidemia. This may result in expensive, disabling complications, and increased use of medical services [9].

DM impacts negatively on working productivity as shown by our data, and indirect costs were larger than direct costs (61% vs. 39%). This negative impact of DM and its complications on productivity has also been shown in other countries with different social and productivity environments. In Argentina, direct costs accounted for 58%. In Brazil, 63.3% related to direct costs and 36.7% to indirect costs [28]. In Mexico, indirect costs represented 56% of the total $7.7 billion (in US dollars as of 2011) spent on diabetes care [29]. Indirect costs represented 59% of diabetes costs in the UK [30]. Direct medical costs accounted for the largest proportion of the economic costs of diabetes in a rural area of China [31].

Regarding the most important component of costs in Brazil, the greatest portion of direct costs was attributed to medication (48.2%) [28]. In the US, the largest component of medical expenditure was hospital inpatient care, accounting for 43% of the total medical costs [26], similarly to Argentina [24]. In our study, laboratory tests represented the main cost relating to people with DM.

Findings of cost predictors reveal some probable axes of inequalities. For example, being male related to higher cost in medication. Women may have reduced access to essential healthcare due to gender biases in power and resources; this can result in increased risk of complications and death [32]. Our results show that income was a predictor of higher cost. People with a higher income may have better access to health services. Another hypothesis for this result is that people with a higher income could be more affected by the illness, with higher requirement for health services [33]. Having a higher level of education was a predictor of low cost in DM; over the last two decades literature has been growing and this illustrates the concept of health literacy as a relevant and influential factor relating to diabetes mellitus [34].

Our findings of high of catastrophic medical spending are consistent with preview studies performed in LMIC [35]. Restricted access to healthcare and medication and the consequent long-term health implications of uncontrolled DM and its complications could be linked to this fact. It generally occur at younger ages in low and middle-income countries compared with high-income countries, which leads to higher loss of healthy life-years in these countries [28].

This study has a number of limitations. Firstly, our study samples excluded undiagnosed cases. Our reported differences and ratios therefore probably overestimate use of medical services per person among persons with undiagnosed DM (and probably with low severity). Secondly, as it is neither possible nor desirable to assign DM experimentally, the study design was observational. However, our case-control design is now the most widely used and accepted approach for measuring the economic and social impacts of diabetes.

In this study we assumed that all those who are temporarily/permanently disabled by DM would have future earnings. The formal sectors are small and the unemployment rate is high and the marginal productivity of labor might be less than the average. This might overestimate the economic burden of DM. In human capital approach values health benefits in terms of production gained due to morbidity (loss of working time), and debility (loss of productive capacity at work). The approach has been criticized for not being consistent with the basic rationale of the economic calculus used in cost-benefit-analysis, and the fact that people are more concerned with preventing premature death, morbidity and debility per se than with preserving productive resources and maintaining future levels of GNP, among other considerations [36]. Besides, the human capital approach could to underestimate costs because it values life using market earnings, thereby excluding retired elderly. It also undervalues life if labor market imperfections exist and wages do not reflect true abilities [10].

Finally, apart from the physical pain associated with diabetes complications, it has other psychological costs. For example, many communities in Africa may be averse to marrying into families with a history of DM, and this may have enormous psychological costs on the families concerned [12].

It is clear that the multi-factorial risk of diabetes, which includes socioeconomic status, sedentary lifestyle and diet, must be viewed from a broader perspective. These data indicate that the cost of DM constitutes a huge burden on society. In the absence of a publically funded healthcare system, these costs are borne almost entirely by individuals who are among the poorest people in the world. The recommended paradigm shift is critical for the development of policies on non-communicable diseases and must integrate diabetes care into the management of the health care system as a whole [37].
